# Estimation of antioxidant, antimicrobial activity and brine shrimp toxicity of plants collected from Oymyakon region of the Republic of Sakha (Yakutia), Russia

**DOI:** 10.1186/0717-6287-47-10

**Published:** 2014-04-01

**Authors:** Babita Paudel, Hari Datta Bhattarai, Il Chan Kim, Hyoungseok Lee, Roman Sofronov, Lena Ivanova, Lena Poryadina, Joung Han Yim

**Affiliations:** Division of Life Sciences, Korea Polar Research Institute, KOPRI, Incheon, 406-840 Republic of Korea; Institute for Biological Problems of Cryolithozone, Siberian Branch of Russian Academy of Sciences, Moscow, Russia

**Keywords:** Antimicrobial, Antioxidant, Brine shrimp, DPPH, Lichen, Moss

## Abstract

**Background:**

Several plants are reported to be produced various biological active compounds. Lichens from the extreme environments such as high altitude, high UV, drought and cold are believed to be synthesized unique types of secondary metabolites than the other one. Several human pathogenic bacteria and fungi have been muted into drug resistant strains. Various synthetic antioxidant compounds have posed carcinogenic effects. This phenomenon needs further research for new effective drugs of natural origin. This manuscript aimed to screen new source of biological active compounds from plants of subarctic origin.

**Results:**

A total of 114 plant species, including 80 species of higher plants, 19 species of lichens and 15 species of mosses, were collected from Oymyakon region of the Republic of Sakha (Yakutia), Russia (63˚20′N, 141˚42′E–63˚15′N, 142˚27′E). Antimicrobial, DPPH free radical scavenging and brine shrimp (Artemia salina) toxicity of all crude extract were evaluated. The obtained result was analyzed and compared with commercial standards. A total of 28 species of higher plants showed very strong antioxidant activity (DPPH IC50, 0.45-5.0 μg/mL), 13 species showed strong activity (DPPH IC50, 5-10 μg/mL), 22 species showed moderate antioxidant activity (DPPH IC50,10-20 μg/mL) and 17 species showed weak antioxidant activity (DPPH IC50 more than 20 μg/mL). Similarly, 3 species of lichen showed strong antioxidant activity, one species showed moderate and 15 species showed weak DPPH reducing activity. In addition, 4 species of mosses showed moderate antioxidant activity and 11 species showed weak antioxidant activity. Similarly, extracts of 51 species of higher plants showed antimicrobial (AM) activity against Staphylococcus aureus and 2 species showed AM activity against Candida albicans. Similarly, 11 species of lichen showed AM activity against S. aureus and 3 species showed AM activity against Escherichia coli. One species of moss showed AM activity against S. aureus. And finally, one species of higher plant Rheum compactum and one species of lichen Flavocetraria cucullata showed the toxicity against Brine shrimp larvae in 100 μg/mL of concentration.

**Conclusion:**

The experimental results showed that subarctic plant species could be potential sources of various biologically active natural compounds.

## Background

Reactive oxygen species (ROS) and reactive nitrogen species (NOS) are accumulated in living organisms during normal metabolic processes and exogenous stimuli such as UV-radiation, stress. ROS, such as superoxide anions (O_2_^–^), hydroxyl radicals (OH), hydrogen peroxide (H_2_O_2_), and hypocholorous acid (HOCl), and NOS such as nitric oxide radical (NO) have been associated with inflammation, cardiovascular diseases, cancer, aging-related disorders, metabolic disorders, and atherosclerosis [1]. ROS are dangerous because they can attack unsaturated fatty acids and cause membrane lipid peroxidation, decreases in membrane fluidity, loss of enzyme receptor activities, and damage to membrane proteins, ultimately leading to cell inactivation and cell death [2]. Living organisms possess a natural defense mechanism that counters the deleterious effects of ROS. Despite the existence of such a mechanism, increasing ROS accumulation over the lifetime of a cell can cause irreversible oxidative damage [3]. Thus, antioxidant agents that can slow or prevent the oxidation process by removing free radical intermediates are desired. Several strong synthetic antioxidants have already been reported [4], however most of them have been proven to be highly carcinogenic [5]. For this reason it has become very necessary to derive antioxidants from natural sources for use as supplements to human health. A wide range of natural compounds, including phenolic compounds, nitrogen compounds, and caretenoids [6–9] have antioxidant properties.

Pathogenic microbes, mainly gram-positive bacteria, pose serious threats to human. *Staphylococcus aureus* especially methicillin resistant *Staphylococcus aureus* (MRSA) species have posed serious threats to human health care settings. New alternatives for combating the spread of infection by antibiotic resistance microbes in future are necessary tools for keeping pace with the evolution of ‘super’ pathogens. Similarly, *Escherichia coli* and *Candida albicans* have also been considered as potential pathogen to human. In this research report we screened the antimicrobial activity of subarctic plants extracts against various bacterial and fungal pathogenic micro-organisms.

Natural products have contributed significantly in development of anticancer drugs [10]. There is an urgent need of searching potential candidates of future anticancer drugs to deal with increasing number of cancer diseases in human beings. Brine shrimp toxic compounds could be a potential candidate of anticancer activity. Therefore, we have used this assay to perform primary screening of subarctic plants extracts for their toxicity.

## Results and discussion

### Antioxidant activity

All the tested plant extracts and the commercial standard (BHA) exhibited DPPH free radical scavenging activities in the concentration dependent manner that could be easily read by a spectrophotometer obtaining a decreased absorbance at 517 nm. BHA is a strong commercial antioxidant compound and the IC_50_ (50% inhibition) of this compound was 4.98 μg/mL in the present experiment. According to observed experimental data in term of 50% inhibition concentration (IC_50_) (Table 1), higher plants showed strong antioxidant activity as compared to lichens and mosses. Among the higher plants, 28 species showed very strong antioxidant activity (IC_50_, 0.45-5.0 μg/mL), 13 species showed strong activity (IC_50_, 5–10 μg/mL), 22 species showed moderate antioxidant activity (IC_50_, 10–20 μg/mL) and 17 species showed weak antioxidant activity (IC_50_ more than 20 μg/mL). *Rhododendron dauricum*, *Dryas grandis* and *Rhodendron redowskianum* showed the strongest antioxidant activity (IC_50_, 0.4-0.6 μg/mL). In case of lichen, 3 species, *Thamnolia vermicularis*, *Peltigera didactyla,* and *Peltigera malacea* showed strong antioxidant activity (IC_50_, 5.2- 6.1 μg/mL), one species of lichen *Peltigera aphthosa* showed moderate and 15 species showed weak antioxidant activity. Similarly, 4 species of mosses showed moderate and remaining 11 species showed weak antioxidant activity.

### Antimicrobial activity

The observed experimental data as shown in Table 1 indicated that 51 species of higher plants showed antimicrobial (AM) activity against *S. aureus*. According to size of zone of inhibition, following four categories of antimicrobial activities against *S. aureus* were obtained. Two species of higher plants, *Empetrum nigrum and Cassiope tetragona* showed very strong AM against *S. aureus* (inhibition zone ≥ 20 mm) and moderate AM activity against *C. albicans* (inhibition zone-10 mm). Three species showed strong (inhibition zone, 15–20 mm), 32 species showed moderate (inhibition zone, 10–15 mm) and 14 species showed weak (inhibition zone ≤10 mm) AM activities. None of the tested higher plants extract showed AM activity against *E. coli* and *A. niger*. Similarly, 11 species of lichens showed various strength of AM activity against *S. aureus*. Two species of lichen, *Alectoria ochroleuca* and *Cladonia verticillata* showed very strong AM activity against *S. aureus* having zone of inhibition 28 and 27 mm respectively. One species, *Sterocaulon paschale* showed strong AM (inhibition zone, 16 mm), 5 species showed moderate and 3 species showed weak AM activity against *S. aureus*. Similarly, 3 species of lichens showed AM activity against *E. coli* out of which 2 species showed moderate and one species showed weak AM activity. Three species of lichens, *Alectoria ochroleuca*, *Cladonia amaurocraea* and *Cladonia verticillata* showed AM activity against both *S. aureus* and *E. coli.* And finally, one species of moss (*Scorpidium scorpioides*) showed AM activity against *S. aureus*. The differences in antimicrobial activities may be due to variation in antimicrobial metabolites among tested samples. AM activity of test samples is species specific. Such results clearly suggest that subarctic plants species are potential source of species specific antimicrobial active compounds.

### Brine shrimp toxicity test

In the present experiment, among the tested 114 plant species, only one species of higher plant *Rheum compactum,* and one species of lichen *Flavocetraria cucullata* showed death of all tested larvae at 100 μg/mL of concentration. The remaining 112 species didn’t show toxicity within 100 μg/mL concentration. In case of berberine chloride, positive control, all the *Artemia* larvae were dead at the 7 μg/mL concentration. In the negative control test all of *Artemia* larvae were alive after 24 h of experiment.

## Conclusion

The observed experimental data clearly showed that most of the tested plants showed potent antioxidant activities *in vitro*. In addition, many higher plants and lichens species showed potent antimicrobial activity against human pathogenic bacteria, *Staphylococcus aureus*, *Escherichia coli* and fungi, *Candida albicans*. In addition, most of the antimicrobial and antioxidant active plants species were not toxic against *Artemia* larvae which could be an indication of being non toxic plant species. In addition, the observed data also clearly showed that several antioxidant and antimicrobial compounds could be obtained from these plants resources. Therefore, further works of isolation and characterization of antioxidant and antimicrobial compounds merit from these plants resources.

## Methods

*Collection and identification of Plants* A total of 114 plant species, including 80 species of higher plants, 19 species of lichens and 15 species of mosses, were collected from Oymyakon region of the Republic of Sakha (Yakutia), Russia (63˚20′N, 141˚42′E −63˚15′N, 142˚27′E) in July 2010. All the plant specimens were identified by using morphological characters.

### Extraction of plants

The collected samples were air dried and grinded completely. Dried and grinded samples of plants specimens (Table 1) were separately extracted in methanol–water (80:20 v/v) at room temperature (RT). The solvent was evaporated in vacuum at 45°C and the extracts were then lyophilized. The crude extracts were stored at −20°C until further use.

**Table 1:** **Antioxidant, antimicrobial activity and brine shrimp toxicity test of plant’s extracts**

Symbol	Species name	DPPH-IC _50_ (μg/mL)	Antimicrobial activity-Inhibition zone (mm) ^1^		
			***S. aureus***	***E. coli***	***C. albicans***
HP-1	*Rhododendron dauricum*	0.45 ± 0.02	-	-	-
HP-2	*Dryas grandis*	0.52 ± 0.03	-	-	-
HP-3	*Rhododendron redowskianum*	0.61 ± 0.02	-	-	-
HP-4	*Dryopteris fragrans*	1.2 ± 0.08	14 ± 1.84	-	-
HP-5	*Saxifraga bronchialis*	1.8 ± 0.07	-	-	-
HP-6	*Aconogonon tripterocarpum*	1.9 ± 0.17	-	-	-
HP-7	*Chamerion angustifolium*	2.1 ± 0.17	-	-	-
HP-8	*Salix pulchra*	2.1 ± 0.15	10 ± 0.9	-	-
HP-9	*Chamerion angustifolium*	2.2 ± 0.06	-	-	-
HP-10	*Betula divaricata*	2.5 ± 0.15	-	-	-
HP-11	*Artemisia vulgaris*	2.8 ± 0.14	-	-	-
HP-12	*Rhododendron lapponicum*	3.1 ± 0.31	15 ± 1.5	-	-
HP-13	*Andromeda polifolia*	3.2 ± 0.26	-	-	-
HP-14	*Vaccinium uliginosum*	3.4 ± 0.14	9 ± 1.06	-	-
HP-15	*Ribes triste*	3.5 ± 0.07	13 ± 1.4	-	-
HP-16	*Comarum palustre*	3.6 ± 0.18	9 ± 1.41	-	-
HP-17	*Salix reptans*	3.7 ± 0.15	-	-	-
HP-18	*Ledum palustre*	3.8 ± 0.27	-	-	-
HP-19	*Rosa acicularis*	3.9 ± 0.19	10 ± 1.8	-	-
HP-20	*Pyrola rotundifolia*	3.9 ± 0.16	-	-	-
HP-21	*Sanguisorba officinalis*	4.1 ± 0.33	12 ± 1.4	-	-
HP-22	*Carex aquatilis*	4.1 ± 0.08	-	-	-
HP-23	*Rubus matsumuranus*	4.3 ± 0.22	11 ± 1.7	-	-
HP-24	*Vaccinium vitis-idaea*	4.7 ± 0.19	8.5 ± 0.51	-	-
HP-25	*Rubus chamaemorus*	4.7 ± 0.38	11 ± 1.5	-	-
HP-26	*Veronica incana*	4.8 ± 0.14	10 ± 1.2	-	-
HP-27	*Pentaphylloides fruticosa*	4.8 ± 0.24	8.5 ± 0.74	-	-
HP-28	*Galium verum*	4.9 ± 0.34	11 ± 1.2	-	-
HP-29	*Cassiope ericoides*	5.1 ± 0.31	11 ± 1.3	-	-
HP-30	*Parnassia palustris*	5.4 ± 0.11	11 ± 0.98	-	-
HP-31	*Dracocephalum palmatum*	6 ± 0.48	10 ± 1.03	-	-
HP-32	*Orostachys spinosa*	6.2 ± 0.19	-	-	-
HP-33	*Salix tschuktschorum*	6.3 ± 0.25	-	-	-
HP-34	*Juniperus communis*	6.8 ± 0.14	11 ± 1.5	-	-
HP-35	*Ranunculus reptans*	7.3 ± 0.23	-	-	-
HP-36	*Thymus pavlovii*	7.5 ± 0.45	11 ± 1.67	-	-
HP-37	*Sparganium hyperboreum*	8.2 ± 0.43	9 ± 0.82	-	-
HP-38	*Saxifraga punctata*	8.2 ± 0.25	-	-	-
HP-39	*Pinus pumila*	9.2 ± 0.64	10 ± 0.85	-	-
HP-40	*Ribes fragrans*	9.5 ± 0.38	9 ± 1.2	-	-
HP-41	*Sedum sukaczevii*	9.8 ± 0.49	-	-	-
HP-42	*Thalictrum foetidum*	10.3 ± 0.31	10 ± 0.83	-	-
HP-43	*Sorbaria sorbifolia*	11 ± 0.88	16 ± 1.36	-	-
HP-44	*Ptarmica salicifolia*	11.1 ± 0.67	9 ± 1.3	-	-
HP-55	*Rheum compactum*	11.2 ± 0.34	18 ± 1.7	-	-
HP-46	*Artemisia lagocephala*	11.6 ± 1.1	9 ± 0.5	-	-
HP-47	*Campanula rotundifolia ssp. langsdorffiana*	11.8 ± 0.71	-	-	-
HP-48	*Veratrum lobelianum*	12 ± 0.72	9 ± 0.9	-	-
HP-49	*Oxycoccus microcarpus*	12.1 ± 0.48	9 ± 0.73	-	-
HP-50	*Beckmannia syzigachne*	13 ± 0.39	10 ± 1.2	-	-
HP-51	*Empetrum nigrum*	15.1 ± 0.9	20 ± 2.2	-	10 ± 0.33
HP-52	*Euprasia hyperborea*	15.1 ± 0.45	12 ± 1.8	-	-
HP-53	*Cassiope tetragona*	15.2 ± 0.76	20 ± 1.8	-	10 ± 0.81
HP-54	*Alopecurus roshevitzianus*	18 ± 1.44	9 ± 0.8	-	-
HP-55	*Chosenia arbutifolia*	18.1 ± 1.25	14 ± 1.4	-	-
HP-56	*Dryas punctata*	18.2 ± 0.91	-	-	-
HP-57	*Achillea millefolium*	19 ± 1.33	14 ± 1.1	-	-
HP-58	*Astragalus frigidus*	19.3 ± 0.97	14 ± 1.7	-	-
HP-59	*Arctophila fulva*	19.5 ± 0.78	12 ± 1.08	-	-
HP-60	*Artemisia jacutica*	19.7 ± 1.18	10 ± 0.9	-	-
HP-61	*Huperzia selago*	19.8 ± 0.59	-	-	-
HP-62	*Equisetum arvense*	19.9 ± 1.39	11 ± 0.7	-	-
HP-63	*Equisetum arvense*	20 ± 1.2	10 ± 0.6	-	-
HP-64	*Dianthus repens*	20.2 ± 1.94	9 ± 1.02	-	-
HP-65	*Cnidium cnidiifolium*	>20	10 ± 0.8	-	-
HP-66	*Menyanthes trifoliata*	>20	12 ± 1.02	-	-
HP-67	*Lycopodium dubium*	>20	-	-	-
HP-68	*Tofieldia coccinea*	>20	-	-	-
HP-69	*Eriophorum medium*	>20	-	-	-
HP-70	*Oxyria digyna*	>20	-	-	-
HP-71	*Equisetum fluviatile*	>20	9 ± 0.83	-	-
HP-72	*Astragalus schelichovii*	>20	10 ± 1.51	-	-
HP-73	*Oxytropis adamsiana*	>20	10 ± 0.71	-	-
HP-74	*Hedysarum alpinum*	>20	10 ± 0.85	-	-
HP-75	*Calamagrostis purpurea ssp. langsdorffii*	>20	-	-	-
HP-76	*Carex saxatilis* ssp. *laxa*	>20	-	-	-
HP-77	*Aconitum macrorhynchum*	>20	9 ± 0.79	-	-
HP-78	*Poa botryoides*	>20	-	-	-
HP-79	*Juncus nodulosus*	>20	10 ± 0.8	-	-
HP-80	*Eleocharis acicularis*	>20	10 ± 0.7	-	-
L-91	*Thamnolia vermicularis*	5.2 ± 0.16	-	-	-
L-92	*Peltigera didactyla*	5.7 ± 0.46	-	-	-
L-93	*Peltigera malacea*	6.1 ± 0.31	-	-	-
L-94	*Peltigera aphthosa*	14.7 ± 1.03	-	-	-
L-95	*Alectoria ochroleuca*	>20	28 ± 2.38	12 ± 0.7	-
L-96	*Asahinea chrysantha*	>20	9 ± 0.69	-	-
L-97	*Cetraria laevigata*	>20	-	-	-
L-98	*Cladonia amaurocraea*	>20	10 ± 0.81	8.5 ± 0.51	-
L-99	*Cladonia arbuscula*	>20	9 ± 0.91	-	-
L-100	*Cladonia gracilis*	>20	-	-	-
L-101	*Cladonia phyllophora*	>20	-	-	-
L-102	*Cladonia stellaris*	>20	9 ± 0.7	-	-
L-103	*Cladonia stygia*	>20	10 ± 0.76	-	-
L-104	*Cladonia verticillata*	>20	27 ± 2.31	10 ± 0.49	-
L-105	*Dactylina arctica*	>20	-	-	-
L-106	*Flavocetraria cucullata*	>20	13 ± 1.1	-	-
L-107	*Flavocetraria nivalis*	>20	14 ± 1.5	-	-
L-108	*Stereocaulon botryosum*	>20	13 ± 0.88	-	-
L-109	*Stereocaulon paschale*	>20	16 ± 1.6	-	-
M-100	*Sphagnum fuscum*	19.7 ± 0.98	-	-	-
M-101	*Loeskypnum badium*	19.8 ± 0.59	-	-	-
M-102	*Hylocomium splendens*	19.8 ± 1.19	-	-	-
M-103	*Polytrichastrum alpinum*	19.9 ± 0.99	-	-	-
M-104	*Scorpidium scorpioides*	>20	11 ± 1.3	-	-
M-105	*Ditrichum flexicaule*	>20	-	-	-
M-106	*Racomitrium lanuginosum*	>20	-	-	-
M-107	*Warnstorfia sarmentosa*	>20	-	-	-
M-108	*Dicranum elongatum*	>20	-	-	-
M-109	*Sphagnum lenense*	>20	-	-	-
M-110	*Sphagnum imbricatum*	>20	-	-	-
M-111	*Rhytidium rugosum*	>20	-	-	-
M-112	*Sphagnum warnstorfii*	>20	-	-	-
M-113	*Sphagnum anogstroemii*	>20	-	-	-
M-114	*Paludella squarrosa*	>20	-	-	-

*HP* Higher Plant, *L* Lichen, *M* Moss, (−)- no activity.

^1^None of the plant extract showed antimicrobial activity against *Aspergillus niger*.

### Biological activity evaluation

Antioxidant, antimicrobial and Brine shrimp lethality tests were performed to evaluate the biological activities of the plant extracts. To estimate the antioxidant potential of plant extracts, DPPH (1, 1-diphenyl-2-picrylhydrazyl) reducing activity was assayed as described previously [11, 12]. Similarly, antimicrobial activity was assayed by disk diffusion method [13] against human pathogenic microorganisms, *Staphylococcus aureus*, *Escherichia coli*, *Candida albicans* and *Aspergillus niger*. Brine shrimp lethality test was performed to estimate the toxicity of sample as described previously [13].
